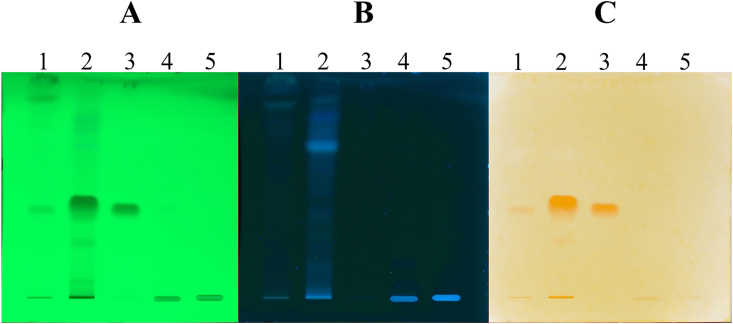# Protozoocidal activity of *Stemona collinsiae* against *Giardia duodenalis*

**DOI:** 10.1016/j.heliyon.2024.e41530

**Published:** 2025-01-02

**Authors:** Khuanchai Koompapong, Supaluk Popruk, Onrapak Reamtong, Tipparat Thiangtrongjit, Sumate Ampawong, Aurapa Sakulpanich, Ruenruetai Udonsom, Kanthinich Thima, Preeyaporn M. Sreepian, Kittiyod Poovorawan, Hirotake Mori, Christen Rune Stensvold, Aongart Mahittikorn

**Affiliations:** a Department of Protozoology, Faculty of Tropical Medicine, Mahidol University, Ratchawithi Road, Ratchathewi, Bangkok, 10400, Thailand; b Department of Molecular Tropical Medicine and Genetics, Faculty of Tropical Medicine, Mahidol University, Ratchawithi Road, Ratchathewi, Bangkok, 10400, Thailand; c Department of Tropical Pathology, Faculty of Tropical Medicine, Mahidol University, Ratchawithi Road, Ratchathewi, Bangkok, 10400, Thailand; d Division of Pharmaceutical Sciences, Faculty of Pharmacy, Thammasat University, Rangsit Campus, Pathum Thani, 12120, Thailand; e Faculty of Medical Technology, Rangsit University, Pathum Thani, 12000, Thailand; f Department of Clinical Tropical Medicine, Faculty of Tropical Medicine, Mahidol University, Ratchawithi Road, Ratchathewi, Bangkok, 10400, Thailand; g Department of General Medicine, Juntendo University Faculty of Medicine, Tokyo, Japan; h Department of Bacteria, Parasites and Fungi, Laboratory of Parasitology, Statens Serum Institut, Copenhagen, Denmark

**Keywords:** *Giardia duodenalis*, Human health, *Stemona collinsiae*, *In vitro* activity, Herbal medicine, Drug development

## Abstract

*Giardia duodenalis* is a major pathogen of the gastrointestinal tract, and infections impact both human health and the economy. The ongoing issues with drug resistance and the side effects of current anti-*Giardia* treatments highlight the urgent need for new therapeutic options. This study focused on investigating the *in vitro* efficacy of crude extracts of *Stemona collinsiae* from Thailand against *G. duodenalis*. *S. collinsiae* was initially extracted with hexane, followed sequentially by dichloromethane, ethanol, and water. The extracts were tested for growth inhibition against *G. duodenalis*. Dichloromethane and hexane extracts were highly active against the parasite after 48 h of exposure, with half-maximal inhibitory concentration (IC50) values of 60.77 μg/mL and 66.66 μg/mL, respectively. Both extracts reduced *G. duodenalis* trophozoite motility and adherence, altered trophozoite architecture, and induced apoptosis. Our findings demonstrate the anti-*Giardia* activity of *S. collinsiae* root extracts, especially those of dichloromethane and hexane. Our results will support the potential development of new and effective medications for the treatment of giardiasis.

## Highlights

• *Stemona collinsiae* was tested for the first time against protozoa.
• Dichloromethane and hexane extracts of *S. collinsiae* displayed high activity against the parasite after 48 h of exposure.
• The crude extracts of *S. collinsiae* affected the motility, adherence, architecture, and induced apoptosis of *Giardia duodenalis* trophozoites *in vitro.*

## Graphical abstract

**Figure:**
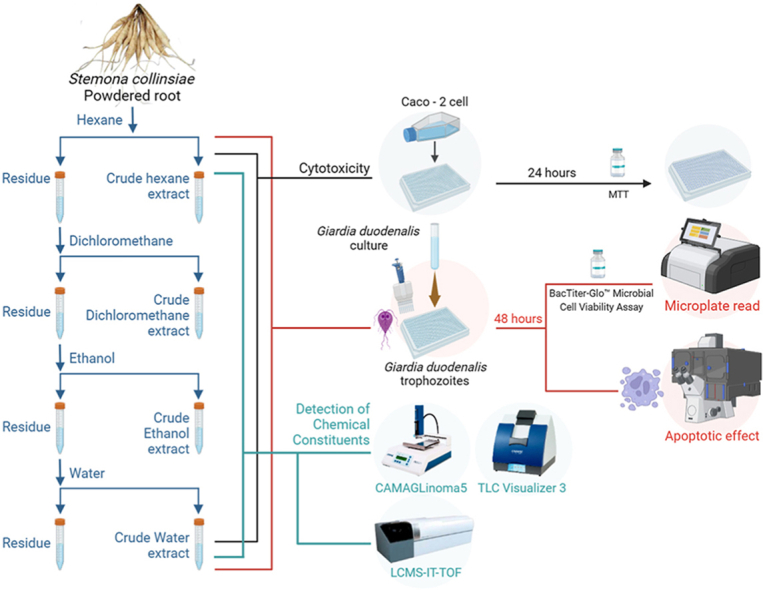

## 1 Introduction

*Giardia duodenalis* (also known as *Giardia intestinalis* or *Giardia lamblia*) is a ubiquitous and robust parasite that is recognized as one of the most important waterborne pathogens worldwide. Common hosts include cats, dogs, and humans. Although 50 %–75 % of giardiasis cases may be asymptomatic [1], its clinical manifestations may include watery diarrhea, nausea, vomiting, and abdominal cramps that are usually self-limiting in immunocompetent individuals. However, severe and prolonged clinical symptoms can occur in immunocompromised individuals, such as those with HIV/AIDS, malignancies, and in those receiving prolonged immunosuppressive drugs. Giardiasis prevalence is estimated to be between 20 % and 30 % in developing nations and between 3 % and 7 % in developed countries [1].

At present, the drugs of choice for treating giardiasis include metronidazole (MET) and other 5-nitroimidazole compounds, such as nitazoxanide and albendazole [2,3]. However, the effectiveness of MET in treating giardiasis has reportedly diminished, with success rates of 60 %–80 % [4]. Cases resistant to treatment have been reported worldwide [5,6]. A study in England found that the failure rate of nitroimidazole treatment for giardiasis increased from 15 % to 40 % within five years [7]. The exact mechanism of metronidazole resistance in *G. duodenalis* is still unknown. Recent studies suggest that genetic changes are highly suspected as a potential cause and are influenced by multiple genetic factors, including copy number variation of the multi-copy flavohemoprotein gene and aneuploidy [8]. Some studies have revealed that both post-transcriptional and post-translational alterations contribute to the reduction of metronidazole-activating enzyme activity [9,10]. MET is not only the drug of choice for *G. duodenalis* but also for *Entamoeba histolytica*, *Blastocystis* spp., and *Trichomonas vaginalis* infections. As a result, drug overuse has led to metabolic adaptation by *Giardia*, resulting in drug resistance or tolerance [11]. Therefore, discovering new compound(s) active against *Giardia* spp. is urgently needed.

High-throughput screening has greatly reduced the time required to develop new therapeutic drugs [12]. However, this technique cannot determine the safety of new drug candidates, especially in long-term clinical practice. For many generations, herbal or traditional medicines have been used to treat certain diseases or illness conditions, especially in tropical countries. While the modes of action and active compounds of many herbal medicinal plants remain unknown, their curative properties and safe usage for certain diseases have long been recognized.

Altogether, 27 species of *Stemona* plants have been discovered throughout Asia and Australia [13]. Many of these have been used as Asian traditional medicines for dermatological and respiratory diseases, as anti-inflammatory drugs, and as biological pesticides [14,15]. Moreover, *Stemona* extracts are known antimicrobials, especially against bacteria such as *Staphylococcus epidermidis*, methicillin-resistant *Staphylococcus aureus,* and *Escherichia coli* [16]. The important phytochemicals in *S. collinsiae* root have been identified as didehydrostemofoline, stemofoline, and hydrostemofoline alkaloids. Of these, didedhydrostemofoline alkaloids are recognized as the major compounds in *S. collinsiae* root and are classified as stemofoline-type derivatives. Their chemical structures consist of an unsaturated lactone ring and an unsaturated butyl side chain attached to a pyrroloazepine nucleus through an oxygen bridge. Didehydrostemofoline is the most potent against diamondback moth larvae [17]. Moreover, *Stemona* alkaloids have also demonstrated anti-inflammatory, acetylcholinesterase inhibitory, and acetylcholine agonist activities [18]. However, the activities of *S. collinsiae* extracts and phytochemicals against parasites have been rarely reported. Currently, the antibacterial mechanism of *S. collinsiae* phytochemical products is not completely understood, and the efficacy of *Stemona* species against pathogenic protozoa has not been evaluated.

Therefore, this study aimed to evaluate the *in vitro* efficacy of *S. collinsiae* extracts against *G*. *duodenalis*. *S. collinsiae* roots were extracted by four extractants: hexane, dichloromethane, ethanol, and purified water. Each extract was then tested for its anti-trophozoite activities against *G. duodenalis* in *vitro* culture. The motility and morphology of the parasite after exposure to *S. collinsiae* root extracts were observed and compared to those exposed to metronidazole (control group). Finally, we determined the cell viability of the parasite using the BacTiter-Glo™ Microbial Cell Viability Assay and an apoptosis detection assay. The results of this study will provide valuable insights into developing new and effective treatments for giardiasis.

## 2 Materials and methods

### 2.1 Plant material

Roots of *S. collinsiae* were harvested from Ubon Ratchathani province, Thailand, between November 2021 and March 2022. The harvested roots were divided into two separate groups. The first group was cultivated in a plantation for the purpose of species identification, while the second group was cleaned, dried, cut, ground, and subjected to extraction using four solvents. The aboveground parts, including the flower inflorescences, as well as the underground parts of *S. collinsiae*, were preserved in a herbarium for plant identification purposes, and voucher specimens (BKF No. 196976) were deposited at the Forest and Plant Conservation Research Office, Department of National Parks, Wildlife, and Plant Conservation. To prepare the roots for extraction, they were initially rinsed with tap water and then dried using an electric fan. The dried roots were chopped into small pieces and further dried in a hot air oven at 55 °C ± 1 °C for 72 h. These chopped, dried roots were then ground using Cross Beater Mill SK 300 (Retsch GmbH, Germany) and sieved with a sieve size of 0.25 mm (Retsch GmbH, Germany). Finally, the powdered roots underwent solvent extraction.

### 2.2 Chemicals

All reagents and solvents used were of analytical grade quality. Hexane, dichloromethane, and ethanol were acquired from RCI Labscan Limited, located in Bangkok, Thailand. Ultrapure water was produced using a Milli-Q® Millipore purification system with 0.22-μm Millipak® express 40 filter (Merck, Germany).

### 2.3 Preparation of *S. collinsiae* root extracts

All extracts utilized in this experiment were derived from a previous study [19]. The powdered root (300 g) was first soaked in hexane and sonicated for 1 h in an Elmasonic E 100H ultrasonic bath (Elma Schmidbauer GmbH, Germany) to expedite extraction. The resulting hexane liquid extract was filtered through Whatman™ grade 1 qualitative filter paper (Cytiva, China), and the resulting filtrate was stored in a glass container shielded from light. Fresh hexane was added to the extraction residue, which was sonicated for an additional 1 h. This sequence of extraction and filtration was repeated until all alkaloids were completely extracted from the residues. The completed extraction was confirmed by thin-layer chromatography (TLC) [19] using Dragendorff's spray reagent composing of solution of basic bismuth nitrate in glacial acid and water and solution of potassium iodide in water [20]. The accumulated filtrates were concentrated with a Rotavapor® R-300 rotary evaporator (BÜCHI Labortechnik AG, Switzerland) at a reduced pressure and a temperature of 40 °C. The hexane crude extract was subsequently dried in a water bath at 70 °C ± 1 °C, transferred to a light-protected, tightly sealed glass container, and stored in a refrigerator at 4 °C. Dichloromethane was added to the residue, which was extracted following the same steps as those used for hexane extraction. When the final filtrate exhibited a negative result on alkaloid testing with Dragendorff's spray reagent, alteration of new solvent from dichloromethane to ethanol was performed with the same process described as above. Likewise, water was final solvent for extraction. The extraction with water was carried out with the same process. The crude water extract was dried by lyophilization.

### 2.4 Cultivation of *G. duodenalis*

The *G. duodenalis* strain used in this study was retrieved from storage in a liquid nitrogen tank and subsequently cultured. This strain was originally isolated from a patient and has been maintained in our laboratory [21]. Trophozoites of *G. duodenalis* were cultured in a modified TYI-S-33 medium, consisting of trypticase, yeast extract, iron, and serum, as described previously [22]. Cultivation was performed *in vitro* under anaerobic conditions at a temperature of 37 °C. After a 24-h incubation period, the growth and viability of the trophozoites were assessed via inverse microscopy (Carl Zeiss Microscopy, LLC, NY). The trophozoites were then harvested during their log growth phase (approximately 2–3 days), and placed on ice for 20 min and then centrifuged at 2000 rpm at 4 °C for 10 min. Following this, a hemocytometer was used to count the trophozoites, which were then prepared for subsequent anti-*Giardia* assays.

### 2.5 Anti-*Giardia* assay

Each crude extract (1 mg) from the roots of *S. collinsiae* was dissolved in 100 % dimethyl sulfoxide (DMSO) and then subjected to a series of twofold dilutions. Metronidazole at 2 μg/mL (Sigma-Aldrich, St Louis, MO) was used as the positive control [23], while 0.25 % DMSO served as the negative control, which had no impact on trophozoites. The blank consisted solely of culture media. Various concentrations of each root extract were tested alongside the controls and blank for cell viability using 96-well microplates. Each well contained a total of 5 × 10^4^ trophozoites with a final volume per well of 100 μL, maintaining a DMSO concentration of 0.25 %. The experiments were performed in triplicate, with plates sealed and incubated at 37 °C for 48 h under anaerobic conditions using 2.5-L Pack-Rectangular Jars (Mitsubishi Gas Chemical, Tokyo, Japan). After 48 h, 100 μL of BacTiter-Glo™ Microbial Cell Viability Assay solution (from Promega Corporation, Madison, USA) was added to every well to measure trophozoite viability through luminescence. The percentage of viable cells in each concentration of crude extract was determined using the following formula:

% cell survival = [(sample luminescence − culture medium luminescence)/non-treated control luminescence − culture medium luminescence] × 100 % inhibition = 100 – % trophozoites that survived

The half-maximal inhibitory concentration (IC50) refers to the concentration of the *S. collinsiae* root crude extract needed to reduce cell growth by 50 %. Different research groups use various criteria to evaluate the effectiveness of plant extracts in inhibiting *Giardia* growth. In this study, we applied the criteria proposed by Amaral et al. [24] as follows: IC50 ≤ 100 μg/mL = highly active; 100 < IC50 ≤ 250 μg/mL = active; 250 < IC50 ≤ 500 μg/mL = moderately active; IC50 > 500 μg/mL = inactive.

### 2.6 MTT assay

For the MTT assay, we used a human-immortalized colorectal adenocarcinoma cell line (Caco-2) (Sigma, Germany) which is widely regarded as a reference for drug absorption and cytotoxicity studies due to its ability to differentiate into enterocyte-like cells. This makes the Caco-2 cell line an excellent model for studying intestinal absorption, permeability, and drug toxicity [25,26]. The Caco-2 cells were cultured in Eagle's minimum essential medium (MEM), which was enhanced with 10 % fetal bovine serum (HyClone, GE Healthcare Life Science, USA), in 96-well plates and maintained for 24 h at 37 °C with 5 % CO_2_. Crude extracts were incorporated into the MEM at concentrations from 10 to 100 μg/mL, and prior to incubation for 24 h, the medium was swapped for 0.5 % DMSO. Each well received 10 μL of a 5 mg/mL solution of 3-(4,5-dimethylthiazol-2-yl)-2,5-diphenyltetrazolium bromide (Panreac AppliChem GmbH, Germany). The plates were then incubated for an additional 4 h at 37 °C in a 5 % CO_2_ atmosphere. The initial solution was replaced with 100 μL of a solvent composed of 4 mM HCl, 0.1 % Nonidet P-40 in isopropanol. Absorbance readings were taken at 590 nm, using a reference wavelength of 620 nm, and cell viability was determined by comparing the absorbance of treated samples to that of the untreated control.

### 2.7 Detection of apoptosis by ethidium bromide/acridine orange (EB/AO) staining

The extracts were assessed for their ability to induce apoptosis in the parasites by staining *Giardia* suspensions with a dye mixture containing 100 μg/mL of ethidium bromide (EB) and acridine orange (AO). Based on their fluorescence colors, cells were classified as follows: green indicated normal cells, orange denoted apoptotic cells, and red signaled necrotic cells. For each group (standard treatment (positive control), non-treatment, and 2 types of extractions), we evaluated the intact cells using fluorescence microscopy in at least ten randomly selected microscopic fields under the high-power field (200× objective)/group or at least a total of 100 *Giardia* trophozoites/group. The percentage of normal or abnormal cell/field among groups were then compared.

### 2.8 Detection of chemical constituents in *S. collinsiae* crude extracts using TLC and liquid chromatography–mass spectrometry (LC-MS)

#### 2.8.1 Preparation of extracts and didehydrostemofoline reference solution

All extracts were prepared at a final concentration of 10 mg/mL for both TLC and LC-MS analyses. Hexane and dichloromethane crude extracts were dissolved in dichloromethane, while the ethanol and water crude extracts were prepared in 70 % methanol. The reference solution consisted of 0.2 mg/mL didehydrostemofoline dissolved in dichloromethane.

The volumes of the extract solution applied to TLC plates and injected for LC-MS analysis were calculated based on IC50 values of dichloromethane and hexane crude extracts.

#### 2.8.2 TLC

Seven microliters of all crude extracts were applied to TLC plates, while 5 μL the didehydrostemofoline reference was applied. The stationary phase consisted of a silica gel GF_254_ TLC plate (Merck, Germany). A Linomat 5 applicator (Camag®, Switzerland) was used for spotting. On the TLC plate, the bandwidth was set at 1 cm. The TLC plate was developed in a tank with a mobile phase mixture of dichloromethane, ethyl acetate, methanol, and 10 % NH_4_OH in a ratio of 70: 25: 5: 1. After development, the plate was dried and examined at UV wavelengths of 254 and 366 nm. Dragendorff's spray reagent was applied to the dried plate, and the presence of alkaloids was indicated by an orange band. Images of the TLC results were captured using a TLC Visualizer 2 (Camag®, Switzerland). The experiment was conducted in triplicate.

#### 2.8.3 LC-MS

Experiments were carried out using a high-performance liquid chromatograph (Model: LC-20ADXR, Shimadzu, Japan) equipped with an UV/VIS detector (SPD-20AV, Shimadzu, Japan) and a mass spectrometer (Model: LCMS-IT-TOF, Shimadzu, Japan). A Dikma® HPLC column Platisil (Length 150 mm × I.D. 4.6 mm, particle size 5 μm) (Cat#. 99501, Ser# 1766742, Dikma Technologies, Inc., CA, USA) with Dikma® easyguard II universal HPLC (DM 62011, Lot No. 3741864) was used for separation. Isocratic elution was carried out at a flow rate of 1 mL/min and a mobile phase consisting of a 55:45 (v/v) mixture of methanol and 1 mM ammonium acetate. Injection volumes were 7 μL for hexane, ethanol and water crude extracts, and 1 μL for the dichloromethane crude extract. Five microliters of the didehydrostemofoline reference was injected. Electrospray ionization was performed in positive and negative modes using nitrogen as desolvation and nebulization gas flowing at 1.5 L/min. The interface and heat block temperatures were maintained at 200 °C. Full-scan mode was performed in the range of 50.00–500.00 *m*/*z*. Events 1, 2, and 3 consisted of the results of the MS positive mode, MS/MS positive mode (precursor ion: 386, CE: 50 %), and MS negative mode, respectively.

### 2.9 Statistical analysis

Descriptive statistics were calculated using SPSS software, version 18.0 (SPSS, Chicago, IL, USA). The data are reported as means with standard deviations. A one-way ANOVA was conducted to compare the normal cell counts per field among negative, positive, and test groups against *G. duodenalis*. Statistical significance was determined at a probability (*p*) value of less than 0.05.

## 3 Results

### 3.1 Cytotoxicity of the extracts against Caco-2 cells and apoptotic effect from the extracts

All four crude extracts were non-cytotoxic to Caco-2 cells at concentrations exceeding 1000 μg/mL at 37 °C for 24 h (Supplementary Fig. 1). However, after 48 h of treatment, the dichloromethane and hexane crude extracts were inhibitory to *G. duodenalis* in a dose-dependent manner, with IC50 values of 60.77 μg/mL (highly active) and 66.66 μg/mL (highly active), respectively (Supplementary Fig. 2). In contrast, the crude extracts from water and ethanol had IC50 values greater than 500 μg/mL. Ethidium bromide/acridine orange staining indicated that dichloromethane and hexane extracts induced apoptosis, as demonstrated by the significantly lower number of intact cells compared to the nontreatment (negative) group (Fig. 1). The apoptotic properties of these two extracts were not significantly different. However, the apoptotic effect of the hexane extract was significantly lower than that of the standard treatment group (positive control). A list of different plant extracts, the plant parts used, and their effects against *in vitro G.* *duodenalis* trophozoites is shown in Table 1.

**Fig. 1:**
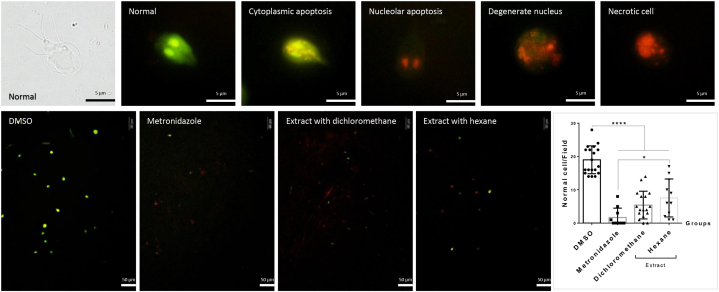
Ethidium bromide/acridine orange (EB/AO) staining of parasites after exposure to different types of *S. collinsiae* root extract compared with standard (positive control)- and nontreatments (negative control): Intact green cells indicate live (normal) cells, diffused orange staining in the cytoplasm, nucleolar necrosis with red nuclei, degenerative nuclei with diffused nucleolar fragmentation with orange/red staining all indicate cytoplasmic apoptosis; and red staining indicates cell necrosis. ∗∗∗∗: *p* value < 0.001; ∗: *p* value < 0.05.

**Table 1:** Medicinal herbs with obvious *in vitro* anti-*Giardia duodenalis* trophozoite effects. Modified from Ref. [27].

Plant name	Part of used	Extraction method	Effect results (trophozoites)	References
*Ageratum conyzoides*	Flower, leaf	Essential oil-hydrodistillation	Degeneration of flagella and ventral disc	[22]
*Citrus aurantifolia*	Peels	Hexane	Growth inhibition	[28]
*Lippia graveolens*	Aerial parts	Aqueous	Morphological change, membrane blebs, internalization of flagella and ventral disc	[29]
*Mangifera indica*	Aerial parts	Aqueous	Reduces viability by 75%	[30]
*Mentha piperita*	Leaves	Methanol, dichloromethane, Hexane	Alteration plasma membrane surface, inhibits adhesion	[31]
*Origanum virens*	Aerial parts	Aqueous	Morphological change, irregular surface, internalization of flagella and ventral disc	[29]
*Pulsatilla chinensis*	Aerial parts	Ethyl acetate, aqueous	Inhibits adherence, precipitates cytoplasm, breaks flagella and ventral disc, membrane blebs	[32]
*Stemona collinsiae*	Root	Dichloromethane, Hexane	Inhibition of motility and adherence	This study
*Thymus zygis*	Aerial parts	Essential oil	Morphological change, irregular surface, internalization of flagella and ventral disc	[29]
*Thymbra capitata*	Aerial parts	Essential oil	Irregular surface, membrane blebs, internalization of flagella and ventral disc	[33]

### 3.2 Detection of chemical constituents in *S. collinsiae* crude extracts using TLC and LC-MS

**Fig. 2:**
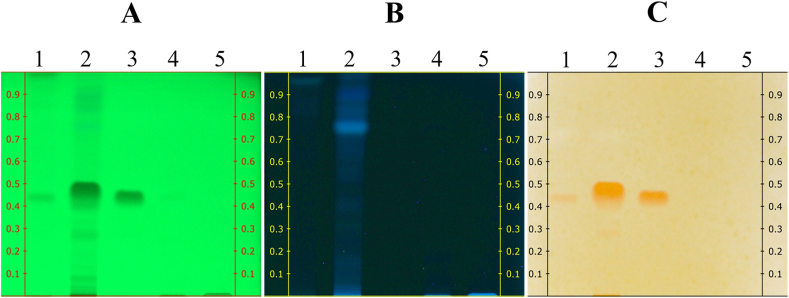
TLC chromatograms of crude extracts of hexane, dichloromethane, ethanol, and water observed under (A) UV 254 nm, (B) 366 nm, and (C) after spraying with Dragendorff's spray reagent for detecting alkaloids. 1 = hexane crude extract; 2 = dichloromethane crude extract; 3 = didehydrostemofoline reference substance; 4 = ethanol crude extract; 5 = water crude extract. The full, non-adjusted, original images of this figure is shown in Supplementary Fig. 3.

The developed TLC plate was observed under UV wavelengths of 254 (Fig. 2A) and 366 nm (Fig. 2B). More quenching bands were observed in the dichloromethane crude extract track (Track 2, Fig. 2A) compared to the other tracks of crude extracts (Tracks 1, 4, 5, Fig. 2A). Under 254 nm, Track 2 in Fig. 2A shows quenching bands at the R_f_ values of 0.08 ± 0.01, 0.28 ± 0.01, 0.76 ± 0.01, and 0.84 ± 0.01. In comparison, the didehydrostemofoline reference substance produced a quenching band at the R_f_ value of 0.44 ± 0.01 (Track 3, Fig. 2A). Quenching bands of didehydrostemofoline in hexane and dichloromethane crude extracts clearly appeared at the R_f_ values of 0.44 ± 0.01 and 0.45 ± 0.01, respectively (Tracks 1 and 2, Fig. 2A). The quenching band of didehydrostemofoline in the dichloromethane crude extract was the most intense, compared to those of the other crude extracts. The quenching band of didehydrostemofoline in the crude ethanol extract track appeared pale black (Track 4, Fig. 2A). No quenching bands were observed in the crude water extract (Track 5, Fig. 2A).

Under 366 nm, the crude dichloromethane extract generated fluorescent bands at the R_f_ values of 0.75 ± 0.03, 0.90 ± 0.01, and 0.99 ± 0.01 (Track 2, Fig. 2B). The crude hexane extract generated fluorescent bands at the Rf values of 0.88 ± 0.01 and 0.96 ± 0.01 (Track 1, Fig. 2A). As didehydrostemofoline does not fluoresce, its band was not detectable at 366 nm.

Spraying the TLC plate with Dragendorff's reagent for detection of alkaloids resulted in the detection of orange bands (corresponding to didehydrostemofoline alkaloid) in the crude extracts of hexane, dichloromethane, and didehydrostemofoline reference, with the R_f_ values of 0.44 ± 0.01, 0.45 ± 0.01, and 0.44 ± 0.01, respectively (Track 1–3, Fig. 2C). This band presented faintly in the crude ethanol extract (Track 4, Fig. 2C) due to the very small amount of didehydrostemofoline in the extract, while it was completely absent in the crude water extracts (Track 5, Fig. 2C).

**Fig. 3:**
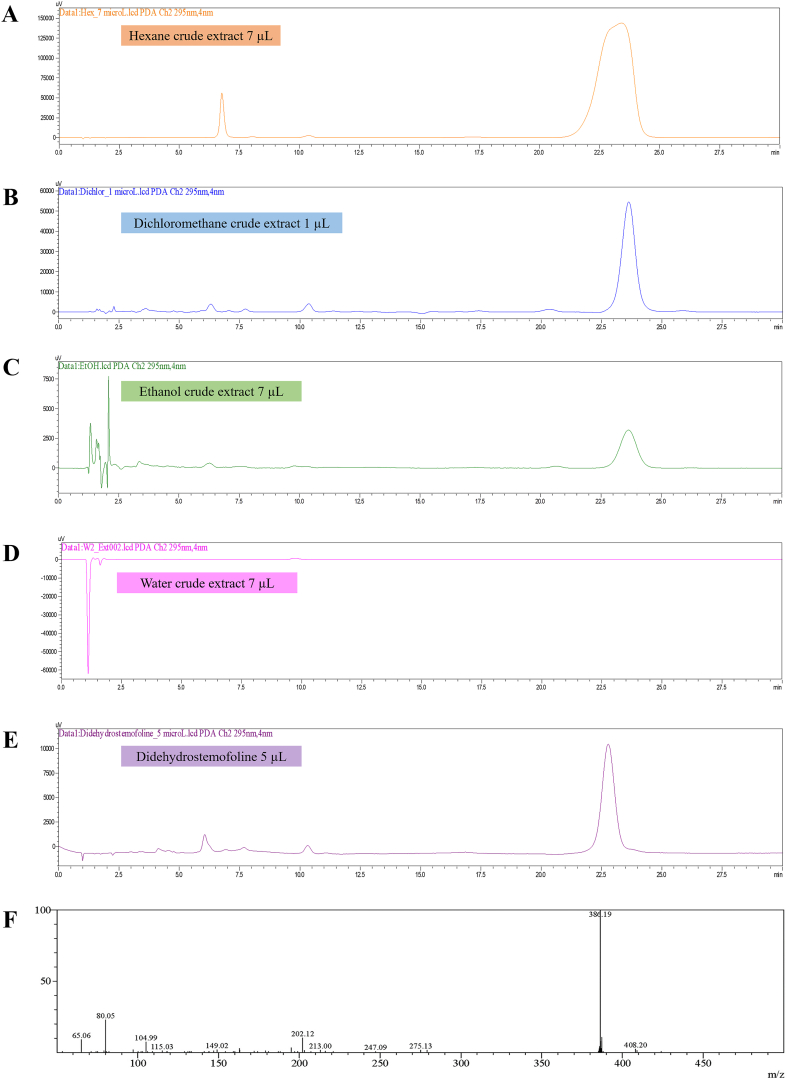
Detection of didehydrostemofoline in the extracts using LC-MS showed LC chromatograms of didehydrostemofoline in crude extracts of (A) hexane, (B) dichloromethane, (C) ethanol, and (D) water crude, and in (E) didehydrostemofoline reference substance, and (F) MS spectrum of didehydrostemofoline peak appearing at 23.47 ± 0.29 min after detection with the LC–MS method.

Didehydrostemofoline in hexane, dichloromethane, and ethanol crude extracts was detectable by HPLC with a UV/Vis detector coupled with mass spectrometry. LC chromatograms of hexane, dichloromethane, and ethanol crude extracts detected didehydrostemofoline at a retention time of 23.47 ± 0.29 min (Fig. 3). Similarly, the MS spectrum of the peak of didehydrostemofoline (MW 386.19 [M + H]^+^) appeared at 23.47 ± 0.29 min (Fig. 3F). In contrast, didehydrostemofoline was not detected in crude water extracts (Fig. 3D). The LC chromatograms of the four crude extracts showed differing contents of didehydrostemofoline in each crude extract (Fig. 3A–D). At an extract concentration of 70 μg/mL, the dichloromethane crude extract contained the highest level of didehydrostemofoline, followed by hexane and ethanol crude extracts. These findings were consistent with the TLC results, although LC-MS analysis was notable for detecting a very small amount of didehydrostemofoline in the crude ethanol extract.

## 4 Discussion

The flagellated parasitic protozoan, *G. duodenalis*, is a causative agent of the food and waterborne diarrheal disease, giardiasis, which can affect humans and other mammals chronically [34]. In the USA, 242 outbreaks of giardiasis were reported from 1971 to 2011 [35]. Cases of resistance of *G. duodenalis* to anti-*Giardia* drugs such as metronidazole, albendazole, and nitazoxanide, which can result in treatment failure, have been documented [11,36].

There have been several studies on herbal extracts against *G. duodenalis* trophozoites and cysts [27]. Various parts of medicinal herbs and extraction methods have shown different results for their anti-*Giardia* effects. Most other studies revealed alterations in the membrane surface of the trophozoite, as well as some degeneration of flagella and the ventral disc (Table 1). The present study explored the potential of *S. collinsiae* root extracts as a source of new drugs by investigating their anti-*Giardia* activity and cytotoxicity. An inhibition of motility and trophozoite adherence was found as well as apoptosis of the trophozoite. To the best of our knowledge, this is the first report on the activity of *S. collinsiae* root against *G. duodenalis*.

The dichloromethane crude extracts of *S. collinsiae* roots were the most potent growth inhibitor of *G. duodenalis* (IC50 = 60.67 μg/mL), followed by that of hexane crude extract (IC50 = 66.66 μg/mL). TLC analysis revealed that the dichloromethane crude extract contained the highest content of the didehydrostemofoline alkaloid and unknown fluorescent substances, followed by that of hexane crude extract. The water crude extracts lacked didehydrostemofoline and unknown fluorescent substances, while that of ethanol crude extract contained only a small amount of didiehydrostemofoline. These results are consistent with those of LC analysis, which found the highest content of didehydrostemofoline alkaloids in dichloromethane crude extracts. Similarly, the dichloromethane crude extract of *S. collinsiae* was the most effective against *Periplaneta americana*, followed by that of hexane crude extract [19,37]. The alkaloids in these extracts modify the acetylcholine pathway, which plays a crucial role in protozoa function [38,39]. Didehydrostemofoline is a major compound in the crude extract of *S. collinsiae* using 70 % ethanol, and it has demonstrated good larvicidal activities against *Musca domestica*, *Chrysomya megacephala* [37], and *Parasarcophaga ruficornis* [14]. This activity is due to the modulation of acetylcholine receptors, inhibition of acetylcholinesterase, stimulation of pupation, and disturbance of insect metamorphosis. A recent study has demonstrated the lethal effects of *S. collinsiae* extracts on the larval stage (L3) of *Gnathostoma spinigerum*, suggesting that didehydrostemofoline is the primary active agent [40]. Similarly, alteration of acetylcholinesterase may disrupt all physiological functions of *G. duodenalis*, leading to parasite death [41]. Therefore, the anti-*Giardia* activity of *S. collinsiae* may be attributed to the presence of didehydrostemofoline alkaloid compounds. Compared to stemofoline and 2′-hydroxystemofoline, didehydrostemofoline is associated with the strongest contact toxicity and insecticidal and growth-inhibitory activities against *Spodoptera littoralis*, which are attributed to differences in the chemical structure of the side chain of these alkaloids [42]. The side chains of stemfoline and 2′-hydroxystemofoline contain a 2′-hydroxy group and a saturated n-butyl group, respectively, which diminishes their insecticidal activity. In contrast, the side chain of didehydrostemofoline contains an unsaturated double bond [42]. Metronidazole is an antiprotozoal drug belonging to the group of 5-nitroimidazole drugs. Its chemical structure consists of an imidazole ring with nitrogen atoms. The nitrogen dioxide group (–NO_2_ group) attached to the imidazole ring serves as both pharmacophore and toxicophore. Metronidazole diffuses into microbial cells, where it changes to a nitroso radical via the oxidation of ferredoxin [43], which normally acts as an electron acceptor from pyruvate dehydrogenase. The radical reacts with DNA to inhibit protein synthesis, and it induces DNA strand breakage, ultimately causing the death of susceptible cells [43,44]. Albendazole or methyl N-(6-propylsulfanyl-1H-benzimidazol-2-yl) carbamate is a benzimidazole type of antihelmintic and antiprotozoal drugs. Albendazole and its combinations are used to treat giardiasis [6,[45], [46], [47], [48], [49]]. Furazolidone belongs to the group of nitrofuran drugs. Anti-*Giardia* drugs such as metronidazole, albendazole, furazolidone, tinidazole, nitazoxanide, quinacrine, chloroquine, and bacitracin are nitroheterocyclic and nitro-containing drugs [50]. Paromomycin, an aminoglycoside antibiotic for treatment of protozoal infections consists of an amine functional group (–NH_2_ group) attached to a heterocyclic ring. It interacts with ribosomal RNA and inhibits protein synthesis [6]. Meanwhile, 4-aminoquinoline in chloroquine is a pharmacophore that binds to Fe(II) [51]. The oxygen bridge or endoperoxide moiety is one of the important pharmacophores in artemisinin [52]. Didehydrostemofoline was the predominant alkaloid in hexane and dichloromethane crude extracts. Its chemical structure consists of a protostemonine skeleton (Stemofoline-type derivatives) comprising a pyrrolo[1,2-α]azepine core attached to two α-methyl-γ-butyrolactone rings [53,54]. An azepine structure and oxygen bridge connects the C-2 and C-8 of its chemical structure, suggesting possible anti-*Giardia* activity. Also, based on the reported nematocidal and leishmanicidal activities including toxicity of rotenoid flavonoids [55] and stilbenoids [56], the unknown fluorescent substances contained in hexane and dichloromethane crude extracts may be rotenoid flavonoids or stilbenoids, which have known anti-*Giardia* activity. The alkaloids and other phytochemicals in the extracts synergized anti-*Giardia* activity, similar to the elimination of insects. The *S. collinsiae* extract contains many active compounds, such as alkaloids, glycosides, terpenes, flavones, and volatile oils. These compounds interfere with the parasite's internal enzymes, disrupt enzyme production centers, block ion passage through cell membranes, and affect the parasite's biological functions [[13], [14], [15],^37^,^42^,^53^]. Therefore, isolated compounds from the extracts should be tested experimentally to identify the actual pharmacological compound and potency of activity.

The mechanism of action of *S. collinsiae* phytochemicals remains unclear. Based on the consistency of our results with those of previous research, as well as the structure-activity relationship of anti-*Giardia* drugs, we speculate that the mechanism of action of *S. collinsiae* phytochemicals possibly involves the disturbance of electron transport. Acetylcholine modulators and acetylcholinesterase inhibitors in *S. collinsiae* phytochemicals may affect the secretion of cytokines that affect the motility of *G. duodenalis*. Dichloromethane and methanol crude extracts have been shown to inhibit the growth of malignant cell lines KB and MCF-7 [57]. A similar anti-proliferative activity may interfere with the cell division of *G. duodenalis*.

This study results suggest that dichloromethane and hexane *S. collinsiae* root extracts are effective against *G. duodenalis in vitro* and might be good candidates for drug development. However, further in-depth studies are needed, including testing the extracts using normal human cell lines and conducting *in vivo* experiments. These studies are necessary to clarify the mechanism of action of the extracts and their phytochemicals, assess the treatment's effectiveness and toxicity, and explore potential clinical applications of the root extracts.

## 5 Conclusion

The results of this study suggest that crude root extracts of *S. collinsiae* are active against *G. duodenalis*, particularly those extracted with dichloromethane and hexane. The dichloromethane and hexane crude extracts of *S. collinsiae* roots affected the motility, adherence, architecture, and apoptosis of *G. duodenalis* trophozoites. Consequently, future studies should aim to identify the active phytochemical compounds in *S. collinsiae* and elucidate their mode of action.

## CRediT authorship contribution statement

**Khuanchai Koompapong:** Writing – original draft, Investigation, Formal analysis, Conceptualization. **Supaluk Popruk:** Writing – review & editing, Software, Resources, Methodology, Investigation, Formal analysis, Conceptualization. **Onrapak Reamtong:** Software, Methodology, Investigation, Formal analysis. **Tipparat Thiangtrongjit:** Methodology. **Sumate Ampawong:** Writing – review & editing, Methodology, Investigation, Formal analysis. **Aurapa Sakulpanich:** Writing – review & editing, Software, Resources, Investigation, Formal analysis. **Ruenruetai Udonsom:** Formal analysis. **Kanthinich Thima:** Resources. **Preeyaporn M. Sreepian:** Formal analysis. **Kittiyod Poovorawan:** Resources. **Hirotake Mori:** Writing – review & editing. **Christen Rune Stensvold:** Writing – review & editing. **Aongart Mahittikorn:** Writing – review & editing, Writing – original draft, Resources, Project administration, Investigation, Formal analysis, Conceptualization.

## Data availability statement

Data included in article/supplementary material is referenced in the article.

## Ethical approval and consent to participate

Not applicable.

## Declaration of competing interest

The authors declare that they have no known competing financial interests or personal relationships that could have appeared to influence the work reported in this paper.

## Figures and Tables

**figs1:**
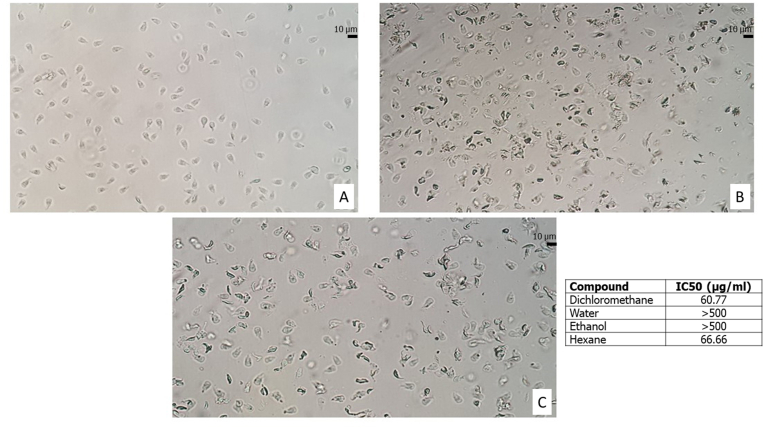


**figs2:**