# Supplementary figures and images for: Protozoocidal activity of Stemona collinsiae against Giardia duodenalis
Source: Heliyon. 2025 Jan 2;11(1):e41530. doi: 10.1016/j.heliyon.2024.e41530 (PMC11761316; doi:10.1016/j.heliyon.2024.e41530)

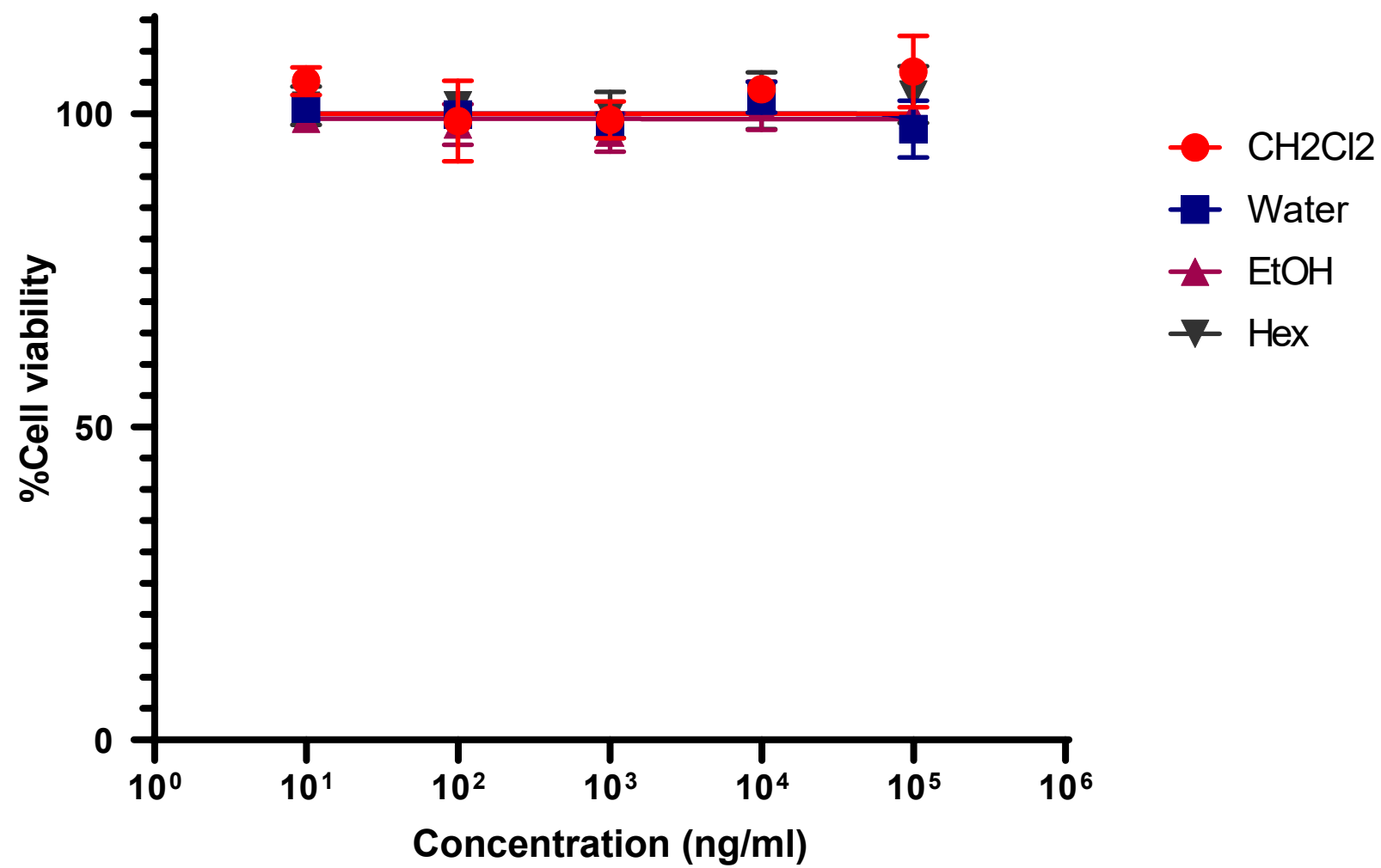

Supplementary Fig. 1 Cytotoxicity of each crude extract on Caco-2 cell line.

Supplement: Multimedia component 1 [file mmc1.pdf]
