# Supplementary material for: Protozoocidal activity of Stemona collinsiae against Giardia duodenalis
Source: Heliyon. 2025 Jan 2;11(1):e41530. doi: 10.1016/j.heliyon.2024.e41530 (PMC11761316; doi:10.1016/j.heliyon.2024.e41530)
Supplement: Multimedia component 1 [file mmc2.pdf]

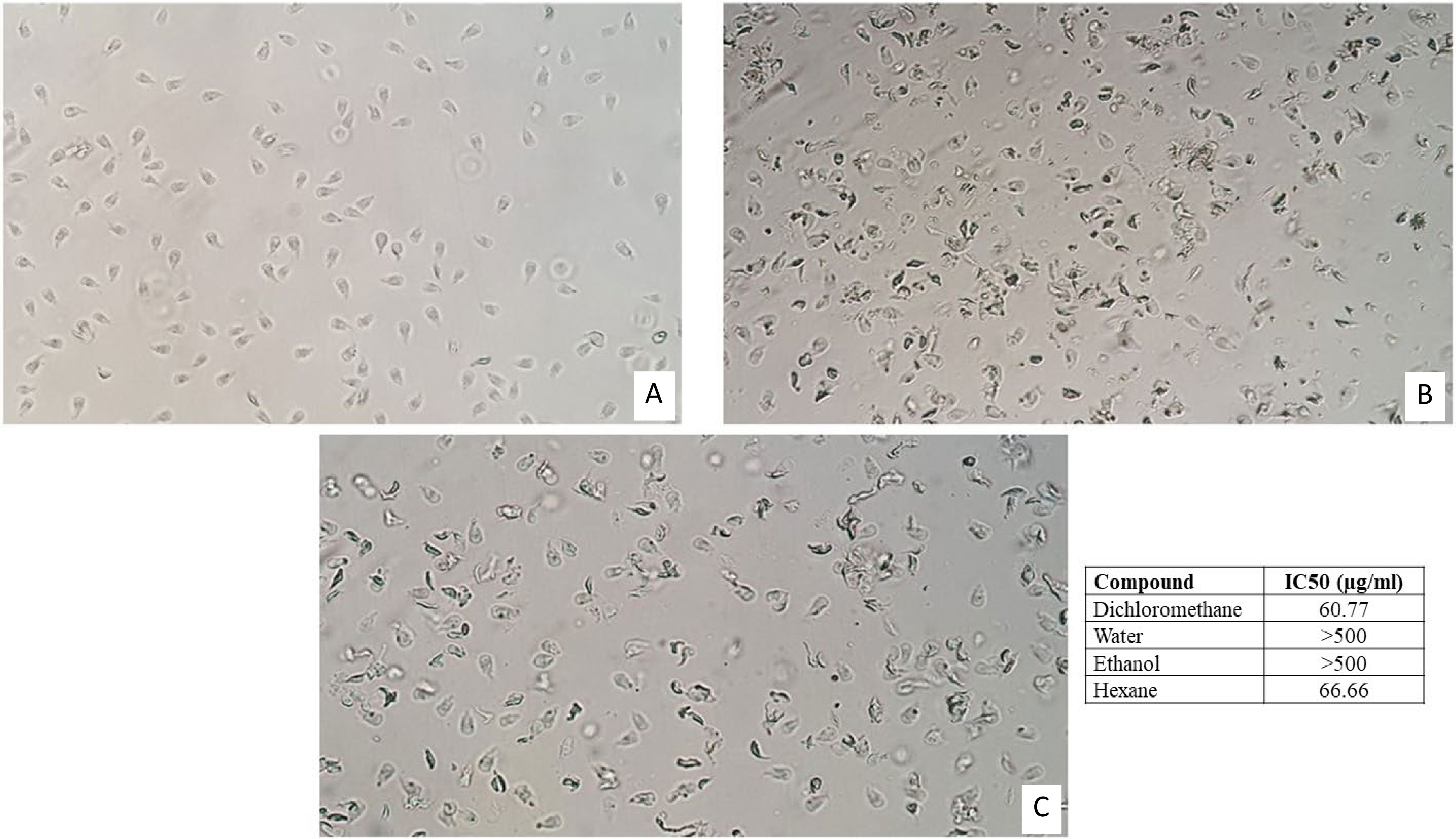

Compound	IC50 (µg/ml)
Dichloromethane	60.77
Water	>500
Ethanol	>500
Hexane	66.66

Supplementary Fig. 2. *Giardia duodenalis* treated with *S. collinsiae* crude extracts after 48 h. A: *G. duodenalis* treated with 0.25% DMSO (negative control); B: *G. duodenalis* treated with crude dichloromethane *S. collinsiae* root extract; C: *G. duodenalis* treated with crude hexane *S. collinsiae* root extract.
